# Up to Standard? A Longitudinal Analysis of Regulatory Compliance in British Zoos

**DOI:** 10.3390/ani16071038

**Published:** 2026-03-28

**Authors:** Chris Lewis, Frankie Osuch

**Affiliations:** Born Free Foundation, Frazer House, 14 Carfax, West Sussex RH12 1ER, UK

**Keywords:** zoos, *ex situ* conservation, zoo inspection, zoo conservation, Zoo Licensing Act, animal welfare, zoo welfare

## Abstract

We analysed the formal inspection reports of 108 licensed British zoos covering three consecutive formal inspections, equivalent to a licensing period, under the Zoo Licensing Act 1981 (ZLA). We examined the compliance of zoos against animal welfare standards, animal escape protocols and engagement in conservation measures, as well as the effects of licence type, zoo association membership, and collection type. Of the 324 inspection reports analysed, 59 (18%) reported that the zoo had passed every assessed question. Failure to undertake the necessary number of escape drills was the most reported area of non-compliance in 134 (41%) of the reports. Across a total of 15,876 welfare assessment criteria, 14,067 (89%) were scored as compliant, but only 83 inspection reports (26%) recorded that the zoo had met all welfare standards. Zoos were commonly found to fall into one of three classes, which predicted their probability of participating in each of the five conservation measures within the ZLA. Farm parks were identified as the collection type performing least well across inspection categories. With British zoos being required to meet new standards from May 2027, we propose a number of changes which could aid inspection consistency and legislative enforcement and drive improvements.

## Simple Summary

Government-appointed zoo inspectors assess the legal compliance of British zoos every three years against the requirements of the Zoo Licensing Act 1981. We analysed 324 reports from 108 zoos to provide the first review of its kind in over a decade. We identify a number of discrepancies within the current inspection process and raise questions in relation to the effectiveness of the process in implementing the requirements of the Zoo Licensing Act 1981, rectifying identified non-compliance, and improving standards in zoos. We discuss our results in the context of the current inspection process and the revised Standards of Modern Zoo Practice for Great Britain which come into force in 2027 and provide recommendations for improvements.

## 1. Introduction

### 1.1. Zoo Licensing in Great Britain

The Zoo Licensing Act 1981 (ZLA) aims to ensure that zoos in Great Britain meet a number of measures listed under s.1A, including high standards of animal welfare, contributing to the conservation of wildlife, promoting public education, preventing escapes and the intrusion of undesired animals into the zoo, and keeping up-to-date records [1]. The ZLA requires zoos to be inspected and licensed to ensure compliance with the Act. Local authorities are responsible for the licensing and organisation of inspections for zoos in their jurisdiction. They are also responsible for enforcement and for deciding whether a zoo retains its licence, has additional conditions linked to required improvements added to its licence, or is issued with a closure notice.

Secretary of State (SoS) zoo inspectors, recruited by the Department for Environment, Food and Rural Affairs (DEFRA) and assigned by the Animal Plant and Health Agency (APHA), are either veterinary surgeons with zoo animal experience or people who have been deemed competent to advise on the management of zoos [1]. These SoS inspectors need to be present for formal inspections (new, periodic, and renewal inspections, plus special inspections when deemed necessary by the licensing authority). The remaining inspectors are people who the local authority consider to be competent for the purpose of inspecting zoos. In contrast to informal or special inspections, formal inspections cover all features of the zoo’s licensing requirements.

Zoos may be granted a dispensation under s.14 of the ZLA if they are considered to be a small collection that keeps less than 200 non-hazardous, non-conservation-sensitive, non-mammalian wild animals or are a zoo with less than 50 hazardous and/or conservation-sensitive animals [1]. (Table S1) Zoos with a dispensation may receive less frequent inspections and are visited by a reduced number of inspectors. Other small animal collections granted a dispensation under s.14(1)(a) are exempt from the requirements of the ZLA entirely. In general, all zoos should be inspected by at least one inspector every three years. A 2012 study found that approximately 84% of licensed English zoos had dispensations, meaning that the minority of zoos were fully licensed [2]. Although DEFRA have previously produced guidelines for when a zoo qualifies for a dispensation, these rarely seem to have been applied [3]. The study concluded that if these guidelines had been strictly followed, dispensations would have applied to approximately half of English zoos [2].

Inspectors are responsible for ensuring that dispensations remain appropriate for the zoo. They are also responsible for issuing recommendations and conditions relating to a zoo’s licence. Whilst there is no compulsory method of completing an inspection report, the ZOO2 form devised by DEFRA is most often used by inspectors [4]. This form was subject to minor adjustments in 2012, in line with updates to the Secretary of State’s Standards of Modern Zoo Practice (SSSMZP), and currently contains 100 questions relating to compliance with the SSSMZP which accompany the ZLA [5]. It allows the inspector to answer “Yes”, “No” or “N/A” and there is an area for comments as well as for recommendations and conditions.

In May 2025, DEFRA published revisions of the SSSMZP, renamed the Standards of Modern Zoo Practice for Great Britain (SMZPGB), which will come into effect in May 2027 [6]. It is anticipated that the zoo inspection form currently utilised by inspectors will be changed to bring it in line with these updated standards, as also indicated in the December 2025 Animal Welfare Strategy for England, in which the Government stated that they will “update any necessary guidance and inspection forms to make it more straightforward for zoo licensing inspectors and local authorities to enforce the new standards by 2027” [7].

### 1.2. Zoo Compliance with Requirements

#### 1.2.1. Animal Welfare

The negative impact of captivity on wild animal welfare is an area which has been extensively investigated in the scientific literature, demonstrating the importance of maintaining high standards of animal welfare in zoos [8,9,10,11]. The ZLA requires zoos to satisfy the physical, psychological, environmental, nutritional and social needs of its wild animals [1]. Broadly, this is done through the provision of food, water, a suitable environment, healthcare, the opportunity to express normal behaviour and the protection from fear and distress, which closely reflect the five needs of animal welfare within s.9 of the Animal Welfare Act 2006 and the Animal Health and Welfare (Scotland) Act 2006 [12,13]. The welfare section of the ZOO2 inspection form relates to these key areas. Welfare, where discussed in this study, is considered in the context of the five needs of animal welfare for continuity.

Draper and Harris [14] analysed 192 inspection reports from different zoos across Great Britain dated between 2005 and 2008 and found that 76% of zoos did not meet all of the animal welfare requirements outlined within the ZOO2 form. More recently, Tyson [15] analysed 564 zoo inspection reports relating to 237 different English zoos, dating between 2008 and 2019, and found that, on average, 66% of zoos did not meet every analysed standard. Although Tyson [15] found a higher percentage of zoos that met all the standards, this does not necessarily indicate that zoos are improving their standards over time. Casamitjana [2] analysed English zoo inspection reports from 2005 to 2011 and found a continuous decrease in the level of compliance with conditions stipulated in the European Council Directive 1999/22/EC (EC Zoos Directive) relating to the keeping of wild animals in zoos, which include animal welfare standards [16]. Instead, the apparent increase in compliance between the Draper and Harris [14] study and the Tyson [15] study is likely due to differences in methodology. Both studies analysed formal ZOO2 inspection reports, but they did not analyse responses to the same questions or sample the same geographic region. Also, Tyson [15] only counted the giving of a “No” answer as non-compliant, whereas Draper and Harris [14] considered a “Yes” answer coupled with a condition for that criterion (“Yes, but” answers, see Section 1.3) to also indicate non-compliance. Despite differences in methodologies, both studies found that the majority of analysed zoos were not compliant with all welfare requirements.

Certain factors may impact compliance. Draper and Harris [14] found that fully licensed zoos are significantly more compliant with standards relating to the provision of animal healthcare compared to zoos with a s.14(2) dispensation. In contrast, Casamitjana [2] found little difference in the level of compliance with animal welfare standards between zoos with a full licence and zoos with a s.14(2) dispensation. Draper and Harris [14] also found that zoos which were members of the British and Irish Association of Zoos and Aquariums (BIAZA) were significantly more likely to be compliant with the provision of healthcare section compared to non-members. However, there was no significant difference between BIAZA members and non-members in terms of compliance with all welfare requirements, despite member zoos being expected to exceed minimum legislative requirements [17].

#### 1.2.2. Conservation Measures

In 2003, the ZLA was amended to include criteria contained in the EC Zoos Directive [16]. The main objective of the EC Zoos Directive was to strengthen zoos’ contribution to the conservation of biodiversity [18]. Whereas issues relating to animal welfare were previously covered by the zoo licensing system, conservation issues were not. Therefore, since 2003, British zoos have had a legal obligation to participate in conservation [1]. Despite this, Casamitjana [2] found that 13% of formal inspection reports indicated that the zoo had failed to meet the conservation conditions of their licence.

The conservation value of zoos remains controversial. Although some claim that zoos continue to play an important role in preserving species, others argue that conservation-sensitive animals are not prioritised for management in zoos across Great Britain and globally [19,20,21,22,23,24,25,26,27,28,29]. The UK Government has previously acknowledged the need to improve the conservation requirements for British zoos [30].

Zoos are currently required to fulfil at least one of five conservation measures outlined in s.1A(a) of the ZLA and question 7.1 of the ZOO2 form [1,4]. The relative ease with which zoos can comply with this requirement has been criticised [24,31]. Despite this, Casamitjana [2] found that 5% of inspection reports indicated that zoos failed to fulfil any of the five measures, with measure (iii) of s.1A(a), “exchange of information in relation to the conservation of wild animals”, being the most common conservation effort undertaken by zoos.

Zoos can actively contribute to the recovery and conservation of species through *ex situ* captive breeding or by contributing to *in situ* conservation projects in the wild [32]. Although it was the least common conservation measure met, Casamitjana [2] found that nearly half (46%) of zoos met question 7.1(v), “the repopulation of an area with, or the reintroduction into the wild of, wild animals”.

#### 1.2.3. Animal Escape Prevention and Protocols

Unlike the requirement to partake in conservation measures, taking precautions to prevent the escape or unauthorised release of animals has always been a requirement of the ZLA [1]. However, it was only after 2012 that a question relating to the carrying out and recording of escape drills (question 8.3) was added to the ZOO2 inspection form [15]. Tyson [15] identified the failure to meet this standard as the most common breach of licence conditions. Of all the analysed reports dated between 2014 and 2019, 36.1% were marked as failing this question.

The level of compliance with this area among British zoos has not been thoroughly explored in the scientific literature, but unlike breaches of conservation requirements, the escape of dangerous animals is often reported in the media. For example, news articles revealed that BIAZA and EAZA member zoos were found to have failed to prevent the escape and the subsequent death of animals in their collection within the decade prior to this study [33,34,35].

### 1.3. Concerns with the Inspection Process

Although BIAZA recognises the UK system of zoo licensing and inspections as one of the most robust in Europe, the system has often faced criticism regarding clarity and continuity [36]. Draper and Harris [14] recommended that the suitability of scoring “Yes” for compliance but then adding a condition for that criterion should be reviewed. The same issue, referred to as the “Yes, but” problem, was also identified by Casamitjana [2]. In 2019, DEFRA issued a notice stating that inspectors must score “No” where there is any non-compliance, however minor [37]. Implementing this would rectify the issue of the “Yes, but” problem and, in theory, make any areas of non-compliance clearer to identify by those reviewing the inspection forms.

Draper and Harris [14] also made numerous recommendations regarding the criteria used to assess animal welfare. Currently, the guidelines focus on resource inputs despite the importance of using animal-based outcomes for assessing the welfare of individual zoo animals [38,39]. Also, the inspection forms provide no indication of the assessment methods used or the number of animals visited [14].

The ability of inspectors to accurately assess welfare has also been questioned. Due to the wide variety of animals kept, zoo inspectors are unlikely to be knowledgeable on all species-specific welfare issues [40]. According to a survey-based study by Greenwood et al. [41], 56% of zoo inspectors believed that the general level of knowledge among inspectors was inconsistent. Also, 8% of zoos declared that they thought inspectors lack sufficient knowledge about particular species.

It has also been suggested that inspectors are not given enough time for in-depth enquiries [40]. Although zoo inspections are carried out over a maximum of three days, the vast majority take place on a single day, with some inspectors visiting multiple zoos in one day [14]. Casamitjana [2] predicted that, on average, inspectors would need over two months for a zoo with a full licence and 12 days for those with a licence dispensation in order for every animal in those zoos to be seen and assessed and suggested that it could result in animal welfare issues going unchallenged year after year. Another criticism is that zoos may be closed to the public during inspections. Visitor presence is known to directly affect animal welfare, meaning that certain aspects of animal welfare in zoos cannot be fully assessed during closure [2,42,43]. At the time of this study, these issues are yet to be publicly addressed by DEFRA.

Introducing stricter conditions and more thorough methods of assessment would only successfully improve the standards of British zoos if such conditions were enforced efficiently. Draper and Harris [14] found that 24% of zoos were reported by inspectors as not compliant with all conditions imposed following previous inspections. Similarly, Casamitjana [2] found that 89% of zoos with formal inspection reports had recurring unsatisfactory issues between 2005 and 2011. Also, Draper, Browne and Harris [44] compared reports from two consecutive inspections of 136 British zoos and found that compliance with animal welfare criteria did not clearly improve between inspections. Other authors have claimed that the ZLA has been effective at improving standards of zoos [45,46].

### 1.4. Objectives

Despite the importance of maintaining high welfare standards for animals in captivity, there are very few studies which investigate how welfare standards in British zoos are formally assessed by inspectors [2,14,15,44]. Even fewer have examined compliance with standards over a full licensing period, or related conservation measures and animal escape prevention and procedures [2,15].

The aim of this study is to investigate the level of compliance of British zoos with standards relating to animal welfare, conservation and animal escapes over three consecutive formal inspections, equivalent to a full licensing cycle. To the authors knowledge, no such study has been completed in over a decade. We also review whether previously identified concerns with the inspection process persist and aim to identify any other issues within the inspection system to inform any review of the inspection process prior to the implementation of the SMZPGB in 2027 [6]. By comparing zoo inspections over a licensing period (three consecutive inspections), we assess whether subsequent inspections improve a zoo’s compliance with the ZLA.

## 2. Methodology

### 2.1. Data Collection

Formal inspection reports were obtained through Freedom of Information (FOI) requests submitted to all local authorities within England, Scotland and Wales, requesting a copy of the most recent formal inspection report for all licensed zoos within their administrative area. The requests were submitted to the local authorities in 2016, 2020, and 2023. Inspection reports were only included in this analysis if they had been completed using the ZOO2 inspection form and the type of inspection was indicated as a renewal or periodical. Some additional reports were obtained from publicly available responses on the website, WhatDoTheyKnow.com or via specific FOI requests to local authorities in 2025, to obtain unsupplied inspection reports. A “zoo” was considered to be any facility licensed under the ZLA; this may include premises referred to as safari parks, aquariums, bird of prey centres, farm parks and traditional mixed-species animal collections, which are required to hold a zoo licence under the ZLA. The “date of last formal inspection” was used to determine whether the three reports were consecutive. All zoos where three consecutive formal inspection reports were obtained, and where the most recent report did not pre-date 2020, were included in our analysis.

The data from the inspection reports were inputted into Microsoft Excel©. The extracted data was cross-checked prior to analysis. The collated data consisted of: (i) the name of the zoo; (ii) the date of inspection; (iii) the BIAZA membership status at time of the analyses as reported on their website; (iv) the EAZA membership status at time of the analyses as reported in their most recently published annual report; (v) the country; (vi) the licence at time of inspection (full or dispensation); and (vii) the zoo inspectors’ responses to Sections 1–5 (animal welfare), Section 7 (conservation, education and research) and Section 8, questions 8.1–8.3 (animal escape prevention and preparedness) from the ZOO2 inspection form. Responses were coded as “Y” for “Yes”, “N” for “No” and “N/A” where reported. Question 1.6 in the ZOO2 form relates to whether visitors undertake any feeding of animals within the zoo; responses were recorded but not included in the analysis due to a “No” response not being representative of substandard animal welfare. In contrast, question 1.6(a) is applicable to welfare provision and the responses were included. Answers to question 7.1(i–v) were also recorded separately from the remainder of Section 7. As with question 1.6, an assessment of “No” by inspectors did not indicate the zoo was non-compliant, but rather that the zoo did not contribute towards that conservation measure. The total number of assessed questions was 59 (49 relating to welfare, 7 for conservation and 3 for escapes).

Within seven inspection reports, the “type of inspection” box (dispensation status) was left blank or it was apparent that the incorrect option had been ticked by inspectors based on information provided within the preamble and the number of SoS inspectors present at the inspection. These reports were recorded as the licence type which best reflected the information contained within those sections of the form.

Nine inspection reports were recorded using the old ZOO2 form which does not include questions 2.3, 4.2 and 8.3. Therefore, for the purpose of analysis, these questions on the nine inspection reports were considered as a pass.

Assessment of whether a zoo was judged to be non-compliant for a particular question followed the methods of Draper and Harris [14] to enable the opportunity to compare with previous findings. A zoo was assessed as being non-compliant where inspectors marked a criterion “No”, or where inspectors marked “Yes” but included in the adjoining comments box reference to a condition or a direction to be added to the zoo’s licence in order meet the necessary standard. These “Yes, but” answers were distinguished from “No” answers, to allow for additional analysis and were coded as “Yc”.

### 2.2. Data Analysis

One provisional BIAZA member was excluded from comparative analysis between BIAZA and non-BIAZA zoos and two “Temporary” EAZA members were excluded from similar comparative analysis.

Data were analysed in R, version 4.3.1. The significance level for all tests was *p* < 0.05.

#### 2.2.1. Analysis of Animal Welfare and Escapes

A Friedman rank sum test was conducted to determine if there was a difference in the number of animal welfare criteria that were scored as substandard between the first, second and third inspections. Tests were also run for BIAZA, non-BIAZA, EAZA and non-EAZA members, and for zoos with and without a licence dispensation. A similar test was conducted for animal escape criteria. Wilcoxon signed-rank tests were run to determine any differences in the number of welfare standards deemed not to have been met between pairs of inspections, with *p*-values adjusted through Bonferroni corrections. Wilcoxon rank sum tests were run to compare the number of substandard criteria between BIAZA and non-BIAZA zoos, EAZA and non-EAZA zoos, as well as those with and without a licence dispensation. Chi-squared tests were performed to establish whether BIAZA membership was associated with the likelihood of specific welfare assessment measures remaining compliant. A negative binomial Generalised Linear Mixed Model (GLMM) was run to investigate the effect of collection type on the number of welfare issues identified by inspectors.

#### 2.2.2. Analysis of Conservation Measures

Changes in the number of zoos meeting each of the five conservation measures within the ZLA were analysed using Cochran’s Q test. Post hoc McNemar’s pairwise comparisons were calculated to determine differences in the number of zoos meeting each measure between inspection pairs, with *p*-values adjusted through Bonferroni corrections. Wilcoxon rank sum tests were carried out to compare the number of conservation measures met by BIAZA and non-BIAZA zoos, EAZA and non-EAZA zoos, as well as those with and without a licence dispensation. Latent Class Analysis (LCA), with 2–4 latent classes, was conducted in order to determine whether zoos can be grouped based on the patterns of conservation measures they meet across inspections, with zoo collection type used as a predictor. A multinomial logistic regression was then conducted in order to test whether zoo collection type predicted latent class membership.

#### 2.2.3. Sensitivity Analysis

Where necessary, sensitivity analysis was undertaken to assess the impact of nine inspection reports where inspectors used the old ZOO2 form which did not include questions 2.3, 4.2 and 8.3. Within the original analysis these missing values were scored as a pass. Sensitivity analysis considered these missing values as substandard scores.

Sensitivity analysis was also undertaken to assess the impact of classifying zoo accreditation status for BIAZA and EAZA based on a zoo’s membership at the time of analysis. Where zoos were not a member of an association at either their first or second inspection, the sensitivity analysis involved a re-run of the statistical tests with these zoos categorised as non-members.

## 3. Results and Discussion

This analysis complements earlier studies, further highlighting concerns with the current zoo inspection process and providing an opportunity for reform ahead of the upcoming changes to zoo standards in 2027 [2,14,15]. Although the revised standards for British zoos contained within the SMZPGB provide a number of upgrades to required welfare requirements in comparison to the current SSSMZP, including greater emphasis and clarification of what zoos “must” do, assessment and enforcement of standards is vital to ensure that zoos are meeting their legislative requirements, including those relating to animal welfare, conservation and public safety measures [5,6].

### 3.1. Types of Zoos and Inspections Analysed

A total of 324 formal inspection reports from 108 zoos were analysed, with a further 207 zoos excluded as the number or date of the inspection reports obtained did not meet the sampling criteria. Reports were dated between 2012 and 2023. The first inspections occurred between 2012 and 2017, second inspections between 2016 and 2021, and third inspections between 2020 and 2023. Of those inspections, 112 (34.57%) were for zoos with a full zoo licence completed under s.10 of the ZLA, and 212 (65.43%) were for zoos with a licence dispensation under s.14 of the ZLA. Seven zoos had their dispensation status removed over the licensing period, likely reflecting either an expansion of the size of zoo or an improvement in the correct application of dispensations in line with guidelines. At present, there is a limited overview contained within Annex C of the Zoo Licensing Act 1981: Guide to the Act’s provisions as what facilities may qualify for a dispensation (Table S1) [3]. It should however be noted that decisions are made on a case-by-case basis, which appears to result in inconsistent interpretations by inspectors. Although this did not result in differences in compliancy scores, it was noted that there were 13 reports from 10 zoos within the study where zoos appeared to have more than the recommended number of animals for a dispensation but were allowed to keep their dispensation status. One zoo consistently did not meet the defined requirements but kept their 14(2) dispensation as they were not likely to expand further in the view of the inspector. In another example, a zoo maintained its dispensation as the SoS inspector felt that a second inspector would not be beneficial. The review and development of assessment criteria in line with the requirements of the new zoo standards, and their incorporation into a section of the inspection form, may aid inspectors and local authorities in evaluating and re-evaluating the dispensation status of zoos on a regular basis to ensure they accurately reflect the operations of the zoo.

The average number of days between inspections was 1164 (range: 370–2380), or approximately three years and two months. Missed periodical inspections led to variations in the time between inspections.

Of the 108 zoos, 49 (45.37%) were BIAZA members, 58 (53.70%) were not members, and one zoo (0.93%) was a provisional member. Of the 49 BIAZA members, 40 were members at the time of their first sampled inspection report, 45 were members at the time of their second inspection and all were members at their third inspection. Also, 23 (21.30%) were EAZA members, 83 (76.85%) were not members and two zoos (1.85%) were “Temporary” members. Of the 23 EAZA members, 21 were members at the time of their first sampled inspection report and all were members at the time of their second and third inspection. The most common type of zoo was general mixed with 46 zoos (42.59%), followed by 18 bird of prey centres (16.67%), 17 aquariums (15.74%), 10 farm parks (9.26%), 7 “other bird” (6.48%), 7 “other” (6.48%) and 3 invertebrate (2.78%). The majority of zoos were located in England (92, 85.19%), with 11 in Scotland (10.19%) and 5 in Wales (4.63%).

### 3.2. General Compliance with Licensing Conditions

The most frequently substandard criterion was question 8.3 (“Are escape drills carried out four times a year, recorded and regularly reviewed (at least two drills should include the escape of a Category 1 animal where present)?”), followed by 3.9 (“Are the animals provided with a documented and maintained programme of preventative and curative veterinary care and nutrition?”) and 3.12 (“Are medicines kept and disposed of correctly?”) (Table 1).

Only 59 inspections (18.21%) passed every assessed question of this study, suggesting that only a small proportion of zoos are fully compliant with the requirements of the ZLA. Given the demonstrable level of non-compliance, it is clear that the whole system of zoo inspections requires review.

On 77 (23.77%) occasions, zoos were determined to have failed to meet all of the additional licensing conditions added to their licence following their previous inspection by being marked as non-compliant for question 12.3 (“Have any additional licence conditions been met?”). Three zoos were scored as “No” at all three of their inspections, 19 zoos on two occasions and 30 at one inspection. While the current study did not investigate whether specific conditions were addressed between inspections, compliance with question 12.3 provides some insight. Concerningly, question 12.3 was the sixth most common fail. At the time of writing, offences are listed under s.19 of the Act and include s.19(2), “If the operator of a zoo fails without reasonable excuse to comply with any condition for the time being attached to a licence for the zoo granted under this Act and held by him, he is guilty of an offence.” [1]. Zoo operators found guilty on summary conviction of an offence under s.19(2) may be fined no more than level 4 on the standard scale (currently £2500) as outlined in s.122 of the Sentencing Act 2020 [47]. However, very few prosecutions have been brought against zoos under the ZLA [15]. A low level of compliance with this question may indicate that the current zoo inspection and licensing process is not efficient at ensuring zoos are meeting the necessary standards and ensuring areas which require improvement are suitably addressed in a timely manner. It also raises further questions as to whether the current penalties for offences under s.19 of the Act are a suitable deterrent for non-compliance [15].

The total number of fails increased with successive inspections for 32 zoos and decreased for nine. However, it is unclear whether the apparent increase in failings for some zoos during the full licensing period is a result of the zoo becoming less compliant with the standards or whether the inspection process has become more stringent. Determining whether zoo standards are getting worse or inspections are getting better at detecting non-compliance is not possible using the data collected for this study. It was noted during analysis that later inspections appeared, on average, more detailed, with inspectors providing more positive and negative comments throughout the inspection forms, than in earlier inspection reports. These changes may be due to the introduction or improvement of zoo inspection training courses and guidance for implementing the ZLA.

### 3.3. Animal Welfare Compliance

#### 3.3.1. Animal Welfare: General Findings

There were 83 inspections (25.62%) where no welfare criteria were deemed substandard. This is similar to the findings of Draper and Harris [14], where 47 (24%) of 192 zoos were assessed as meeting all the animal welfare standards and Draper, Browne and Harris [44] where 35 (26%) of 136 zoos met all welfare criteria at the first inspection. However, Draper, Browne and Harris [44] also found that a higher percentage of zoos (32%) met all welfare criteria at the second inspection (dated between 2009 and 2012). In contrast, the number of zoos that met all welfare criteria in this study decreased with each subsequent inspection (39 at first inspection (36.11%), 27 at second (25%), 17 at third (15.74%)).

Across all zoos, the majority of welfare assessments were deemed compliant at each inspection, with 445/5292 (8.41%) welfare requirements deemed to be substandard by inspectors for the first inspection, 536/5292 (10.13%) at second inspection and 828/5292 (15.65%) at the third inspection. In total, 14,067/15,876 (88.61%) of the welfare assessment criteria were scored as compliant. The mean number of welfare criteria (Sections 1–5) per zoo considered by inspectors to be substandard was 4.12/49 in the first inspection, 4.96/49 in the second inspection and 7.67/49 in the third inspection. Across all welfare assessments, the number marked as compliant (89.69%) is higher than the 83% reported by previous research [14].

There was a significant difference in the number of welfare criteria that were scored as substandard between the first, second and third inspections (Friedman rank sum test, χ (2) = 21.84, *p* < 0.01). After Bonferroni correction, there was a statistically significant increase in the number of welfare standards deemed not to have been met between the second and third inspection of zoos (Wilcoxon signed-rank test, V = 1323.5, *p* < 0.01) and the first and third (Wilcoxon signed-rank test, V = 1187, *p* < 0.01), but there was no significant difference between first and second inspections. All outcomes remained unchanged following sensitivity analysis (Table S2).

The percentage of welfare requirements deemed to be substandard in the first inspections (8.41%) is also similar to Draper and Harris’ [14] findings that 9% of questions were graded as substandard and Draper, Browne and Harris’ [44] findings that 7.9% of individual criteria were assessed as substandard at the first inspection and 6.2% at the second inspection. However, the percentage of substandard questions at the second (10.13%) and third (15.65%) inspections in the current study is higher. While Draper and Harris [14] analysed reports dated between 2005 and 2008 and Draper, Browne and Harris [44] analysed reports dated between 2005 and 2012, the first inspection reports in the current study were dated between 2012 and 2017. As there appears to be a similar level of compliance with welfare standards despite the four-to-twelve-year gap between these inspections, this may suggest that the increasing level of assessed non-compliance observed in the current study began post-2012.

While compliance with animal welfare criteria generally decreased with each inspection, it is unclear whether the welfare experienced by the animals concerned changed throughout the licensing period, or whether conditions remained broadly similar but were deemed substandard in later inspections. The increase in substandard criteria could be due to an increase in knowledge and training of inspectors, an increase in inspector scrutiny, or other unknown factors. This study scored “Yes, but” as “No”, so it is unlikely that the decrease in compliance was due to the DEFRA-instructed change in form completion. More research is needed to determine whether the apparent decrease in compliance with animal welfare standards is a result of inspections becoming more stringent and/or better informed when it comes to enforcing the legislation.

Thorough assessment of zoo inspection forms is hampered by the current ZOO2 form not requiring inspectors to record which animals within a zoo have been assessed as part of the inspection. Such a requirement would provide greater clarity, especially for determining the severity of any criteria marked “No”. As per DEFRA’s [37] notice to inspectors, “No” should be scored for any observed non-compliance; however, at present it is unclear, unless a detailed comment is provided, whether a criterion marked “No” relates to one animal, several animals of a species, or several species within the zoo. Despite this, almost a quarter of inspection reports in this study indicated that zoos did not provide an environment which met the physical, psychological and social needs of at least one animal within their care (Table 1). As highlighted by Tyson [15], it can be argued that offences relating to animal welfare should be treated more harshly than those which are not, and that s.19 of the Act should be amended to include a fine of no more than level 5 on the standard scale (which, at the time of publication, can be unlimited), for breaches relating to s.1A(c)(i), “providing each animal with an environment well adapted to meet the physical, psychological and social needs of the species to which it belongs” [47]. The weighting of particular questions may also be worthy of consideration to ensure timely and efficient rectification measures.

#### 3.3.2. Animal Welfare: Zoo Association Membership

There was no significant difference between BIAZA and non-BIAZA zoos in the mean number of welfare non-compliance identified by inspectors across their three inspections, nor between EAZA and non-EAZA zoos. This complements Draper and Harris [14] who found that BIAZA membership did not indicate higher overall conformity with standards of welfare. However, whilst this study analysed the assessed animal welfare standards from Sections 1–5 of the ZOO2 form collectively, Draper and Harris [14] also analysed animal welfare sections on the inspection report separately and did find that BIAZA members performed significantly better than non-members in Section 3 (provision of animal healthcare) and Section 5 (provision of protection from fear and distress).

At each additional inspection, the number of welfare criteria reported to be non-compliant by inspectors increased for accredited and non-accredited zoos. There was a significant difference in the number of welfare criteria that were scored as substandard between first, second and third inspections for both BIAZA- (Friedman rank sum test, χ (2) = 12.435, *p* < 0.01) and non-BIAZA-accredited zoos (Friedman rank sum test, χ (2) = 9.686, *p* < 0.01), as well as EAZA- (Friedman rank sum test, χ (2) = 12.816, *p* < 0.01) and non-EAZA-accredited zoos (Friedman rank sum test, χ (2) = 12.190, *p* < 0.01). All outcomes remained significant following sensitivity analysis (Table S2).

The findings of this study indicate that accredited zoos are less likely to remain compliant with individual welfare standards than non-accredited zoos. There was a significant association between accreditation status and zoos remaining compliant with welfare standards between their second and third inspection, but not their first and second. In their second and third inspection, BIAZA zoos were significantly less likely to remain compliant with an individual welfare criterion than non-BIAZA zoos (Fisher’s exact test, OR = 0.805, 95% CI 0.674–0.961, *p* = 0.01). There was no significant difference between BIAZA members and non-member zoos in rectifying identified issues where criteria remained substandard between the first and second inspection, or second and third inspection.

In contrast, Draper, Browne and Harris [44] found that BIAZA members were more likely to meet the standards on both inspections and less likely to have criteria remaining substandard, as there were significant differences between BIAZA members and non-members in the number of criteria remaining compliant between the first and second inspections and the number of criteria remaining substandard between the first and second inspections. The differences in findings may be explained by the different methodologies; Draper, Browne and Harris [44] accounted for whether the zoo was a BIAZA member during that inspection year, whereas the current study only recorded each zoo’s membership at the time of analysis. However, a sensitivity analysis of this study’s findings rendered the same result (Table S2). Another possible explanation could be the differences in dispensation type between BIAZA and non-BIAZA members (see Section 3.3.3) and the type of inspection they undergo, however this would require further investigation.

#### 3.3.3. Animal Welfare: Collection and Dispensation Type

There was a significant difference in the number of welfare criteria that were scored as substandard between the first, second and third inspections for zoos with a full licence (Friedman rank sum test, χ (2) = 8.536, *p* = 0.01), but not for zoos with a licence dispensation (Friedman rank sum test, χ (2) = 0.693, *p* = 0.707).

Zoos with a full licence had significantly more welfare criteria marked as substandard than zoos with a licence dispensation (Wilcoxon rank sum test, W = 766.5, *p* < 0.01). This differs from previous research [2,14]. This may be explained by more thorough inspections of zoos with full licences under s.10 of the ZLA or the larger number of animals increasing the probability that at least one area of non-compliance is identified.

On average, collection types categorised as “other bird” had the fewest number of substandard welfare criteria (M = 3.29 per inspection report) followed by aquariums (M = 3.41), with farm parks having the highest (M = 7.53). A negative binomial GLMM found no significant effect of collection type on the number of welfare issues identified by inspectors. However, farm parks had 3.7 times more welfare criteria deemed substandard than the baseline (Table S3). The interaction between zoo collection type and inspection number was not significant, indicating similar trends across inspection periods. Due to a low sample size, the collection type “invertebrate” was omitted from the GLMM analysis. Previous research also found that farm parks were judged to have the lowest compliance with welfare standards than other collection types [14]. As suggested by Draper and Harris [14], farm parks may score lower for welfare compliance as they may have less resources or expertise for keeping wild animals given their main focus on keeping domesticated species. While we recommend that different report forms should be used for different types of zoos, it is important that all zoo types should be required to meet consistent welfare standards. Welfare standards should be species-specific rather than dependent on the type of collection or licence they are held under. The additional detail and requirements contained within the revised SMZPGB may help standardise welfare requirements across zoo types and assist collections such as farm parks in determining whether they have the necessary expertise and resources to meet the requirements for particular species before procuring them.

#### 3.3.4. Discrepancies in Animal Welfare Assessments

During the collection and analysis of the data, general observations were made. These included the identification of discrepancies between whether the same comment was allocated as a condition, a recommendation or mentioned just as a comment within different inspections. Not only does this impact on the results of investigations, but it may also indicate that the standards to which zoos are held may vary depending on the inspector(s). Examples of these discrepancies include the practice of defrosting frozen meat in the fridge and the need to have an independent person on the ethical review committee.

Other differences in inspector approaches have also resulted in different scores for zoos that were non-compliant for the same criteria. For example, one zoo’s reptile exhibit was not padlocked and could potentially be opened by a member of the public, but as the issue was rectified during the inspection, no condition was given, whereas another zoo was given an immediate condition to securely lock the gates for an animal paddock.

Another example involved some zoos being marked as compliant and others as non-compliant under question 3.2 (“Do animals on display to the public appear in good health?”) for exhibiting animals with visible ailments that were receiving veterinary treatment. In one instance, question 3.2 was marked as “Yes”, with the comment that one animal had a skin infection that was being treated and a reptile appeared to have a bone infection. Another zoo was marked as “No” as an animal had an ulcer that was receiving appropriate veterinary attention. A further zoo was marked as non-compliant for this question on two of their reports due to ailments that were a result of the animals’ lives prior to being rescued by the current facility, but which were now under appropriate veterinary care.

Some reports also contained comments relating to potentially serious welfare concerns which did not appear to result in the issuing of a condition or recommendation. For example, one report stated that two primates reportedly died from “stress” when isolated, and another zoo’s report commented that the zoo had attempted to rehome a fish whose enclosure size was potentially too small, but that may require euthanasia if their skin lesions prevent rehoming.

### 3.4. Assessment of Conservation Measures

#### 3.4.1. Conservation Measures: General Findings

The number of conservation measures met by a zoo ranged from zero to five out of five (Table 2). Approximately half of all inspections reported zoos participating in four or five of the conservation measures (50.93%). The mean number of conservation measures met per zoo was 3.21 (first inspection), 3.39 (second inspection) and 3.18 (third inspection), with a mean of 3.26 across all inspections. The net change in conservation measures carried out by zoos between their first inspection and their third inspection ranged from +4 to −4, with 42 meeting the criteria for the same number of measures, 34 increasing in the number of measures met and 32 meeting fewer measures.

There were 20 occasions (6.17%) (Table 2) where inspectors determined that a zoo was not meeting any of its conservation requirements under s.1A of the ZLA, involving 13 zoos, with five zoos failing to meet the requirements twice and one zoo being adjudged to have failed to meet any of the requirements throughout the entire six-year licensing period.

The conservation measure met by the most zoos was 7.1(iii) (“the exchange of information relating to the conservation of species of wild animals”). Measure 7.1(v) (“where appropriate, the repopulation of an area with, or the reintroduction into the wild of, wild animals”) was conducted the least (Table 3), complementing previous findings [2]. This was the case across all three inspections. There was a significant increase in the number of zoos found to be conducting research from which conservation benefits accrue to species of wild animals for 7.1(i) (Cochran’s Q (2) = 7.60, *p* = 0.022) (Table S4). There was no significant difference in the number of zoos meeting the other four conservation measures across inspections (Table S5). Post hoc McNemar’s tests found no significant difference in the number of zoos meeting each measure between pairs of inspections following Bonferroni corrections.

The ever-reducing number of zoos contributing towards the reintroduction of wild species is not necessarily surprising when considering the logistical challenges involved. Limited capacity, locational difficulties and prioritisation of visitor expectations are fundamental challenges for the majority of zoos. An analysis of 1863 articles included in the North American Conservation Translocations database from 1974 to 2013 revealed that zoos provided animals for only 14% of translocated species [48]. Similarly, zoos and aquariums provided animals for just 49 of 243 (20%) conservation-translocation projects reported in five Global Re-introduction Perspectives publications [49]. Therefore, in recent decades, zoos appear to have shifted their focus away from being “arks” for conservation breeding towards a much broader conservation mission, particularly involving public education, networking and scientific research [50]. The decrease over time in the number of zoos meeting the reintroduction conservation measure and the increase in those meeting the broader conservation research measure may therefore reflect the shifting missions of modern zoos. The significant increase over time for conservation research may also be due to an increasing number of options for achieving this measure, as reflected by the revised SMZPGB which provide the greatest number of examples for how zoos can meet this measure in comparison to the other four conservation measures [6].

There were inconsistencies among different inspectors in their approach to conservation-related questions, including different interpretations for whether conservation actions, such as repopulation, were being met (see Section 3.4.4). Previously, zoos were deemed to contribute to reintroduction projects if they provided animals for release, funding, staff, expertise, equipment or project coordination [49]. Some participating zoos claimed to contribute to reintroduction conservation projects by providing education and research resources, food, training, habitat management, press coverage or transportation [49]. It may be that zoos also use these indirect contributions to meet the criteria of question 7.1(v) during their inspection. There have also been calls for zoos to measure and evaluate their contribution to conservation [51]. These varying interpretations may also have contributed to the decrease in zoos meeting this conservation measure over time in the view of inspectors. This requirement has been clarified in the revised standards, stating any repopulation or reintroduction “must be part of a recognised project and must follow appropriate guidelines” [6].

Overall, inspectors adjudged the conservation output from most zoos to be appropriate in the context of resource availability. However, 51 reports (15.74%) from 36 different zoos (33.33%) failed question 7.5 (“Are the conservation efforts adequate for the resources of the collection?”). This is a similar result to previous research, where 11% failed, despite this earlier investigation using inspection reports dated between 2005 and 2011 (between one and eighteen years older than the reports used in the current investigation) [2]. With a third of all zoos being adjudged by inspectors to have failed to meet minimum conservation contribution criteria during the study period, it would suggest that more needs to be done to ensure British zoos are meeting their legally required conservation obligations.

#### 3.4.2. Conservation Measures: Zoo Association Membership

BIAZA member zoos scored as meeting significantly more of the conservation measures than non-BIAZA members (Wilcoxon rank sum test, W = 9370, *p* < 0.01). This significant difference was also the case for EAZA-accredited zoos compared to non-EAZA-accredited zoos (Wilcoxon rank sum test, W = 3837.5, *p* < 0.01). These findings were also significant following sensitivity analysis (Table S2).

This may be due to zoos that are members of zoo associations typically having larger budgets and networking connections rather than any direct impact of accreditation on conservation contribution. In the current study, conservation contribution could only be measured by compliance with the relevant criteria on the inspection form. While assessment is likely to be improved by the greater detail on conservation measures in the revised standards, future studies could aim to assess the degree to which meeting the measures required during inspections translates into genuine conservation impact.

#### 3.4.3. Conservation Measures: Collection and Dispensation Type

Zoos with a full licence scored as meeting significantly more of the conservation measures compared to those with a licence dispensation (Wilcoxon rank sum test, W = 14,819, *p* < 0.01). Again, this may indicate that the larger zoos are more likely to have the income and networking connections to achieve more of these measures.

**Figure 1:**
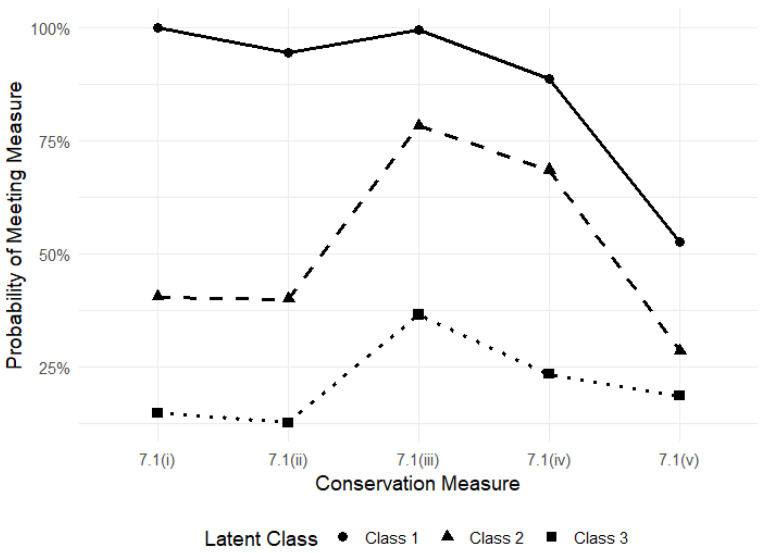
A 3-class model using Latent Class Analysis for predicting the probability of a British zoo undertaking the five conservation measures under s.1A of the ZLA.

On average, zoos categorised as “other bird” were judged to be meeting the most conservation measures as required by the ZLA (M = 3.95/5), followed by traditional general mixed zoos (M = 3.75/5). In contrast, farm parks undertook the least (M = 1.03/5). In order to statistically assess compliance with conservation measures, we used an LCA 3-class model. This was selected as the best fit (BIC = 1780.681; AIC = 1671.040) based on fit indices and interpretability (Table S6). The model indicated 51.44% of zoos belonged to Class 1, 33.74% to Class 2 and 14.82% to Class 3. Zoos within Class 1 were highly likely to meet four conservation measures under the ZLA and may also engage in the reintroduction or repopulation of wild animals: Class 1 zoos demonstrated high probabilities (>0.85) of meeting measures 7.1(i)–7.1(iv) and a moderate probability (>0.50) of meeting measure 7.1(v). Class 2 zoos were likely to participate in two conservation measures, particularly the exchange of information relating to the conservation of species of wild animals and the breeding of wild animals in captivity: Class 2 zoos showed moderately low probabilities (0.25–0.45) of meeting measures 7.1(i), (ii) and (v), but moderately high probabilities (0.65–0.85) of meeting measures 7.1(iii) and (iv). Class 3 zoos were unlikely to meet any of the conservation measures under the ZLA, but of all five were most likely to participate in the exchange of information relating to the conservation of species of wild animals: Class 3 zoos had generally low probabilities (<0.40) across all measures (Figure 1).

For all collection types, the odds ratios (OR) of a collection being in one latent class compared to the baseline could not be reliably estimated due to small group sizes for certain collection types. Similarly, large confidence intervals were obtained due to particular collection types never occurring in a specific class or occurring in very small numbers. Farm parks were exclusively classified in Class 3 and invertebrate collections were exclusively classified in Class 2. Aquariums and bird of prey centres were significantly more likely to be found in Class 2 than Class 1 (Table S7).

The majority of British zoos have a licence dispensation and are not members of a zoo association. The results suggest that, particularly for these zoos, inspections do not drive increased conservation activity. The finding that farm parks are consistently judged to meet fewer conservation measures compared to other zoo types may suggest that the current inspection form is more suited to assessing larger general mixed zoos and that the conservation measures on the form are not necessarily relevant to all zoo types (see Section 3.6). The 2022 consultation on revised standards for British zoos included proposals for different conservation measures depending on the zoo’s size (small, medium and large) in addition to baseline standards for all zoos [52]. Based on the evidence of this study, the originally proposed method of assessing facilities by specific standards that are more suitable to their potential capabilities should be reconsidered in order to facilitate greater conservation engagement by particular facility types that are currently adjudged to be participating in very little conservation activity. At present, within the SMZPGB, an additional note indicates that, “The required conservation measures will be assessed by the zoo inspector(s) in proportion to the size of the zoo and its resources,” however there is no standardised procedure contained within the standards to assist inspectors in making such an assessment, risking inconsistent and subjective assessments by inspectors [6].

#### 3.4.4. Discrepancies in Conservation Assessments

It was noted during the current study that inspectors offered different interpretations for whether a zoo met conservation criteria. For example, one zoo was marked “No” for 7.1(v) with the comment that the zoo’s work to rehabilitate injured birds should be commended but it does not constitute repopulation or reintroduction. Another zoo was marked “Yes” for the same question due to the zoo’s treatment of wild birds and for facilitating migration. Other zoos were also judged to meet this conservation measure simply through the provision of habitats for free-roaming wild animals on site. In a review of the implementation of the ZLA, one of the six interviewed zoo inspectors raised concerns about the inconsistent assessment of conservation and education requirements [53]. Although the requirement for all zoos to meet at least one of five conservation measures has been retained in the revised standards, the actions zoos can take to meet these standards has been clarified through the addition of notes under each measure indicating examples of how zoos can meet each criteria and examples of actions which do not (such as the captive breeding of non-threatened species) [6].

### 3.5. Animal Escape Prevention and Protocols Compliance

The majority of inspections judged zoos to be prepared for incidents where animal escapes could occur. In 64 (19.75%) of the reports analysed, inspectors felt zoos did not have satisfactory measures in place to prevent animal escapes as they were non-compliant for question 8.1. There was no significant difference in the number of escape criteria that were scored as substandard between first, second and third inspections. The statistical outcome remained unchanged following sensitivity analysis, indicating that the finding was robust.

The question that was most commonly found to be substandard was 8.3 (“Are escape drills carried out four times a year, recorded and regularly reviewed (at least two drills should include the escape of a Category 1 animal where present)?”) with non-compliance across 134 reports (41.36%) (Table 1). This issue was identified in 63.33% of all farm park inspections and 50.00% of all bird of prey facility inspections. Tyson [15] found that 36.1% of all reports between 2014 and 2019 were marked as failing this question, indicating that compliance with this requirement has not significantly improved. Although direct comparisons are hindered by a number of the forms within Tyson’s [15] study not including question 8.3, the findings of this study appear to be similar. The requirement was added to the zoo inspection after 2012; and it has been suggested that the scale of non-compliance may indicate a lack of awareness among zoos that this requirement was added and suggests that alterations to zoo requirements are not always reflected by changes in British zoo management strategies [15]. While it may not directly impact animal welfare, consistent failures to meet this standard are still concerning, especially given there were at least 11 reported incidents in 2024 involving animals escaping from a British zoo, including those listed as Risk Category 1 in the SSSMZP or on the invasive non-native species list for England and Wales [54]. It is likely that more incidents of animal escapes went unreported, particularly if the animal did not escape beyond the zoo perimeter and therefore did not require the zoo to notify its local licensing authority [5]. One inspection report in the current study even reported that two mammals escaped on the day of the inspection, one of whom was recaptured on the day. Given the high level of non-compliance, the importance of frequent escape drills should be frequently emphasised to zoos by local authorities at informal inspections.

### 3.6. Factors Limiting the Efficiency of Zoo Inspections

In addition to discrepancies in the assessment of animal welfare conditions and conservation measures, general discrepancies were identified within the completion of inspection reports. Multiple inspection reports acknowledged the 2019 “Yes, but” change in the preamble yet still included a suggested licence condition with a “Yes” answer. “Yes, but” answers were more frequent prior to 21 January 2019, the date that DEFRA issued a notice which asked inspectors to score “No” in any cases of non-compliance, with “Yes, but” answers occurring 549 times out of a possible 11,023 answers (4.98%) and appearing on 111 of the 170 reports (65.29%). For inspections completed after this date, “Yes, but” answers were noted in 195 out of a possible 10,010 (1.95%) answers and appeared on 73 of the 154 inspection reports (47.40%). Despite the DEFRA notice, it appears some inspectors are still utilising the “Yes, but” response, as evidenced by almost half of all inspection reports analysed after the notice containing a “Yes, but” answer. In addition to “Yes, but” responses, several instances were noted where the inspector selected “No” but the report form suggested that they did not issue any condition related to the non-compliance. On a few occasions, “N/A” was recorded when “No” would have likely been more appropriate. For example, one zoo scored “N/A” for 7.4 and 7.6 as they currently had no educational facilities or research efforts.

The ability of inspectors to conduct thorough assessments over a single day should be urgently investigated, with only 29 inspections (8.95%) conducted over two or more days. For one zoo, the inspector noted that too much of the inspection day was spent accessing the zoo’s records. Given that there is often a large amount to cover and the importance, from both an animal welfare and public safety perspective, that all elements are thoroughly inspected, the time allocated for individual zoo inspections should be carefully considered. This will become even more critical with the introduction of the SMZPGB in 2027. Unlike inspections of other establishments such as food businesses and retailers, formal inspections occur with prior notice (of at least 28 days), providing operators the chance to prepare and an opportunity to ensure inspectors see the zoo in the best way possible [1]. While it may not be possible for all aspects of the zoo to be inspected unannounced, such as off-show areas and records and details of education, conservation and research activities, the accurate inspection of animal enclosures is potentially compromised by the prior notice. The conditions observed during the inspection may not reflect the typical daily conditions, especially given the amount of time between formal inspections. While informal inspections may be unannounced in the years where formal inspections are not legally required, these do not commonly occur with a SoS inspector present and are frequently utilised by licensing authorities to check on a zoo’s progress against conditions attached to the zoo’s licence and to address recommendations from prior formal inspections [3]. Therefore, it is also important for as much of the zoo as possible to be inspected by SoS inspectors unannounced. Any desk-based assessment of zoo records, as well as a discussion of the findings from the unannounced inspection between inspectors and zoo personnel, should then take place within a set period after the unannounced inspection.

The suitability of the inspection form in assessing the wide range of facilities also requires review. Aquariums had, on average, fewer reported failings compared to general mixed zoos. This may further indicate that the criteria on the current form is best suited to inspecting more traditional zoos than other zoo types, as it lacks aquarium-specific questions but includes questions which would be unlikely to apply to an aquarium. For example, although the form provides opportunities for assessment under “any other environmental parameters”, the form lacks questions specifically about water flow, water quality, turbulence, overcrowding and intra-tank predation. It is important for inspection forms to be as specific as possible to ensure that important facility-specific criteria have been assessed. Particular sections of the ZOO2 form also remain extremely lean and prevent in-depth assessment, for example, Sections 4 (opportunity to express most normal behaviour) and 5 (protection from fear and distress) of the form are covered by two and three questions respectively, far fewer than other welfare-related sections [4]. The provision of a separate form for particular types of collections was put forward as far back as 2011 as a number of local authorities and inspectors indicated that the current inspection form did not reflect the different types of animal collections [53].

The SMZPGB have been formulated, in part, “to assist zoo inspectors in assessing the standards of animal husbandry, animal welfare and many other factors relevant to the operation of a zoo” [6]. The new standards may improve understanding of and compliance with the requirements through clearer, more detailed standards, with conservation measures being further clarified to aid assessment. However, some elements, such as assessing the size category of the zoo, are still left to the discretion of the inspector. As with assessing licence dispensation suitability, the development of assessment criteria in line with the requirements of the new zoo standards, and their incorporation into a section of the inspection form, may aid inspectors and local authorities in evaluating the size category of a zoo. Such a criteria was proposed within the draft SMZPGB [52].

Enforcement may also be improved, as the new standards specify when to issue a direction to the Licence Holder. The discrepancies in the issuing of recommendations and conditions identified in this study may also be addressed by the addition of more specific standards, such as for defrosting meat and the ethical review process including at least one independent member (see Section 3.3.4). However, consistency in the assessment and enforcement of conditions will need to be monitored to identify if any other common discrepancies occur. Greater ongoing research on this topic is required as the SMZPGB are implemented to ensure zoos are being inspected against the new standards in a reliable and uniform manner by the inspectorate.

### 3.7. Limitations of the Current Study

This study examined zoo inspection reports over the stated period, which rely on zoo inspectors providing simple answers to questions that typically reflect complex issues. As such, the analysis may not truly reflect the differences in compliance between zoos. In some cases, a single issue may result in a zoo failing multiple relevant criteria; in others, a zoo may have multiple failings which may have resulted in only one box being marked as non-compliant.

At the time of inspection, some zoos in this study were closed to the public due to the coronavirus (COVID-19) pandemic, which would have impacted the ability to assess certain criteria. Other zoos were also closed for the winter season, with one zoo inspection containing a comment that this made several aspects of the inspection easier but meant that inspectors were unable to assess some important aspects of visitor management and interaction. In order for inspectors to obtain a broad impression of a zoo’s operation, a significant proportion of any formal inspection should be carried out during visitor opening hours.

The sample size for this study was approximately one-third of all licensed British zoos. Data collection was hindered by local authorities not holding or sharing the correct information, resulting in 207 zoos being excluded from the analysis. Local authorities are strongly encouraged to send copies of completed formal inspection reports to the APHA, with a 2011 DEFRA-commissioned review of the implementation of the ZLA finding that 59% of surveyed local authorities “always” sent these to APHA (formerly, AHVLA) or the Welsh Government [53]. Currently, this is only a mandatory requirement for inspection reports relating to local-authority-owned zoos [3]. In light of the findings of this study, it is recommended that inspection reports for all licensed zoos should be copied to APHA or the Welsh Government, as appropriate, in order to aid future research through the establishment of a central repository as well as improve inspections through the increased opportunity for APHA to provide feedback to inspectors. Such a recommendation was also suggested over ten years prior to this study in 2011 [53].

## 4. Conclusions

The effectiveness and impact of any legislation rely on its implementation and enforcement [55]. The SMZPGB will only drive meaningful change if the standards are accompanied by a thorough and robust reform of the licensing and inspection process. At present, and as highlighted by previous researchers, questions remain as to whether the ZLA is being effectively enforced and whether local authorities are making full use of the powers available to them under the legislation to ensure swift rectification of licensing breaches. The zoo inspection process should be designed not just to assess compliance on each inspection day, but also to assist zoos in continually improving their standards of animal welfare, conservation, public safety and all other areas dictated by the ZLA.

The implementation of the SMZPGB in May 2027 provides a crucial opportunity to revise the inspection process to address the issues identified in this study. To that end, the following recommendations should be considered:

- Criteria or a scoring system for assessing whether a zoo should be granted a licence dispensation under s.14 of the ZLA should be reviewed and made publicly available.
- As zoo collections regularly change their species composition, the ZOO2 form should contain a section designed to assess the aforementioned licence dispensation criteria at each inspection to determine whether a zoo meets, or continues to meet, the conditions for a licence dispensation, including space for an explanatory note by the inspector.
- A similar criterion, as outlined within the draft SMZPGB during the consultation process, should be developed to assess and record which size category a zoo sits within.
- Any zoo which is categorised as “medium” or “large” should be formally inspected over a minimum of two days, with at least one day being carried out during visitor opening hours.
- As zoos become larger, they should be subject to additional specific conservation criteria. Consideration should also be given to judging different collection types by conservation criteria that are more specific to their operation, including those which operate as *bona fide* sanctuaries.
- The submission of copies of formal inspection reports by local licensing authorities to APHA should be mandatory for all licensed zoos to aid future research and increase the opportunity for APHA to provide feedback to inspectors.
- The initial phase of a formal inspection should be carried out when the zoo is open to the public, without prior notice to the zoo operator, to ensure that observations are reflective of the typical daily operations of the facility. Any desk-based assessment of zoo records, which form part of the periodical inspection process, should take place within a set period following the unannounced inspection. Informal inspections should always be unannounced.
- Consideration should be given to creating different inspection forms for different types of licensed zoo, particularly aquariums and other specialist collections, as recommended by previous reviews [14,52].
- The ZOO2 form should include a section specifying which animals/enclosures within a zoo have been assessed as part of the inspection as well as recording the start and end time of this particular part of the inspection.
- Inspection forms should be reviewed regularly, and inspectors should be required to undertake refresher training where evidence of “Yes, but” answers, inconsistent grading of particular questions and other discrepancies are detected, in order to increase consistency among inspectors and inspections.
- A review of s.19 of the ZLA and its implementation by local authorities when offences have been identified should be conducted, and should include consideration of the actions taken by local authorities, whether or not powers under s.19 were exercised, and whether those actions resulted in conditions being met by the specified deadline, as well as the addition of offences under s.1A(c).

## Figures and Tables

**Table 1:** The top ten substandard criteria with the number of fails (“No” and “Yes, but” answers) and the number and percentage of all 324 reports that were marked as non-compliant for that criterion.

Criterion Number	Criterion Category	Question	Number of Fails (% of Reports)
8.3	Public safety	Are escape drills carried out four times a year, recorded and regularly reviewed (at least two drills should include the escape of a Category 1 animal where present)?	134 (41.36%)
3.9	Provision of animal healthcare	Are the animals provided with a documented and maintained programme of preventative and curative veterinary care and nutrition?	97 (29.94%)
3.12	Provision of animal healthcare	Are medicines kept and disposed of correctly?	86 (26.54%)
2.1	Provision of suitable environment	Are the animals provided with an environment well adapted to meet the physical, psychological and social needs of the species to which they belong?	78 (24.07%)
1.3b	Provision of food and water	Are supplies of food and water prepared hygienically?	77 (23.77%)
12.3	Compliance check including licence conditions	Have any additional licence conditions been met?	77 (23.77%)
3.10	Provision of animal healthcare	Is there a system for the regular review of clinical and pathological records?	69 (21.30%)
3.15	Provision of animal healthcare	Are *post mortem* examination arrangements satisfactory?	68 (20.99%)
2.8a	Provision of suitable environment	Is the standard of maintenance adequate for the buildings?	67 (20.68%)
2.3	Provision of suitable environment	Are there satisfactory measures in place to safely confine the animals?	66 (20.37%)

**Table 2:** Total number of conservation measures met by each zoo (*n* = 108) under s.1A of the Zoo Licensing Act 1981 (ZLA) within their first, second and third inspection.

Total Conservation Measures Met	Inspection 1	Inspection 2	Inspection 3	Total
0	7 (6.48%)	4 (3.70%)	9 (8.33%)	20 (6.17%)
1	12 (11.11%)	9 (8.33%)	9 (8.33%)	30 (9.26%)
2	13 (12.04%)	16 (14.81%)	15 (13.89%)	44 (13.58%)
3	24 (22.22%)	20 (18.52%)	21 (19.44%)	65 (20.06%)
4	23 (21.30%)	30 (27.78%)	29 (26.85%)	82 (25.31%)
5	29 (26.85%)	29 (26.85%)	25 (23.15%)	83 (25.62%)

**Table 3:** Total number of zoos (*n* = 108) adjudged by inspectors to be meeting each conservation measure under s.1A of the ZLA within their first, second and third inspection.

Conservation Measure	Inspection 1	Inspection 2	Inspection 3	Total
(i) Research from which conservation benefits accrue to species of wild animals	65 (60.19%)	77 (71.30%)	76 (70.37%)	218 (67.28%)
(ii) Training in relevant conservation skills	69 (63.89%)	71 (65.74%)	67 (62.04%)	207 (64.69%)
(iii) The exchange of information relating to the conservation of species of wild animals	89 (82.41%)	93 (86.11%)	87 (80.56%)	269 (84.06%)
(iv) Where appropriate, breeding of wild animals in captivity	76 (70.37%)	81 (75%)	77 (71.30%)	234 (73.13%)
(v) Where appropriate, the repopulation of an area with, or the reintroduction into the wild of, wild animals	48 (44.44%)	44 (40.74%)	36 (33.33%)	128 (40%)
Total	347/540 (64.26%)	366/540 (67.78%)	343/540 (63.52%)	1056/1620 (65.19%)

## Data Availability

The data that support the findings of this study are available from the corresponding author upon reasonable request.

## Supplementary Materials

The following supporting information can be downloaded at https://www.mdpi.com/article/10.3390/ani16071038/s1, Table S1. Zoo dispensation and exemption criteria; Table S2. Outcomes of sensitivity analysis; Table S3. Effect of collection type on the number of animal welfare criteria reported to be non-compliant by inspectors using a negative binomial Generalised Linear Mixed Model (GLMM); Table S4. Difference in the number of zoos meeting conservation measures under s.1A of the ZLA across inspections; Table S5. Difference in the number of zoos meeting each conservation measure under s.1A of the ZLA across different paired inspections; Table S6. Model fit comparisons for Latent Class Analysis; Table S7. Latent Class Analysis to estimate likelihood of participation in different conservation measures by zoo collection type.

## References

[1] UK Government (1981). Zoo Licensing Act. https://www.legislation.gov.uk/ukpga/1981/37.

[2] Casamitjana J. Inspecting Zoos: A Study of the Official Zoo Inspection System in England from 2005 to 2011. Manchester, UK, 2012. https://www.freedomforanimals.org.uk/Handlers/Download.ashx?IDMF=442125de-c8ac-41b5-bd9d-3bd73e99fbb7.

[3] DEFRA Zoo Licensing Act 1981: Guide to the Act’s Provisions. London, 2012. https://assets.publishing.service.gov.uk/media/5a79dbfce5274a18ba50f569/zoo-licensing-act-guide.pdf.

[4] DEFRA ZOO2 Inspection Report form London, 2013. https://assets.publishing.service.gov.uk/media/5a7f6e3bed915d74e622a68b/zoo2-inspection-report-form.pdf.

[5] DEFRA Secretary of State’s Standards of Modern Zoo Practice. London, 2012. https://assets.publishing.service.gov.uk/media/5a78ce01ed915d042206578f/standards-of-zoo-practice.pdf.

[6] DEFRA Standards of Modern Zoo Practice for Great Britain. London, 2025. https://assets.publishing.service.gov.uk/media/697393b8d345446f8ce71ea5/Standards_of_modern_zoo_practice.pdf.

[7] DEFRA Animal Welfare Strategy for England. London, 2025. https://www.gov.uk/government/publications/animal-welfare-strategy-for-england/animal-welfare-strategy-for-england.

[8] Clubb R., Mason G. (2003). Captivity effects on wide-ranging carnivores. Nature.

[9] Morgan K.N., Tromborg C.T. (2007). Sources of stress in captivity. Appl. Anim. Behav. Sci..

[10] Mason G.J. (2010). Species differences in responses to captivity: Stress, welfare and the comparative method. Trends Ecol. Evol..

[11] Pierce J., Bekoff M. (2018). A Postzoo Future: Why Welfare Fails Animals in Zoos. J. Appl. Anim. Welf. Sci..

[12] UK Government (2006). Animal Welfare Act. https://www.legislation.gov.uk/ukpga/2006/45/contents.

[13] Scottish Parliament (2006). Animal Health and Welfare (Scotland) Act. https://www.legislation.gov.uk/asp/2006/11/section/19.

[14] Draper C., Harris S. (2012). The Assessment of Animal Welfare in British Zoos by Government-Appointed Inspectors. Animals.

[15] Tyson E. (2021). Licensing Laws and Animal Welfare: The Legal Protection of Wild Animals.

[16] European Council Council Directive 1999/22/EC of 29 March 1999 Relating to the Keeping of Wild Animals in Zoos. 1999/22/EC Brussels, 1999. https://eur-lex.europa.eu/legal-content/EN/TXT/HTML/?uri=CELEX:31999L0022.

[17] BIAZA (2026). Join BIAZA. https://biaza.org.uk/join-biaza.

[18] European Comission Meeting Report: First Member State and Stakeholder Meeting (18 February 2020). Supporting Better Implementation of the Zoos Directive. Brussels, 2020. https://ec.europa.eu/environment/nature/legislation/zoos/stakeholdermeetings/pdf/Zoos_1st%20meeting%20report_final_updated.pdf.

[19] Conway W.G. (2011). Buying time for wild animals with zoos. Zoo Biol..

[20] Gippoliti S. (2012). Ex situ conservation programmes in European zoological gardens: Can we afford to lose them?. Biodivers. Conserv..

[21] Fa J.E., Gusset M., Flesness N., Conde D.A. (2014). Zoos have yet to unveil their full conservation potential. Anim. Conserv..

[22] Biega A.M., Lamont M., Mooers A., Bowkett A.E., Martin T.E. (2019). Guiding the prioritization of the most endangered and evolutionary distinct birds for new zoo conservation programs. Zoo Biol..

[23] Draper C. (2017). Zoo Licensing and Inspection: Using Legislative Requirements to Assess Animal Welfare and Conservation in British Zoos.

[24] Born Free Foundation Conservation or Collection? Horsham, 2021. https://www.bornfree.org.uk/resource/conservation-or-collection/.

[25] Marešová J., Frynta D. (2008). Noah’s Ark is full of common species attractive to humans: The case of boid snakes in zoos. Ecol. Econ..

[26] Frynta D., Lišková S., Bültmann S., Burda H. (2010). Being Attractive Brings Advantages: The Case of Parrot Species in Captivity. PLoS ONE.

[27] Conde D.A., Colchero F., Gusset M., Pearce-Kelly P., Byers O., Flesness N., Browne R.K., Jones O.R. (2013). Zoos through the Lens of the IUCN Red List: A Global Metapopulation Approach to Support Conservation Breeding Programs. PLoS ONE.

[28] Martin T.E., Lurbiecki H., Joy J.B., Mooers A.O. (2014). Mammal and bird species held in zoos are less endemic and less threatened than their close relatives not held in zoos. Anim. Conserv..

[29] Kerr K.C.R. (2021). Zoo animals as “proxy species” for threatened sister taxa: Defining a novel form of species surrogacy. Zoo Biol..

[30] DEFRA Action Plan for Animal Welfare. London, 2021. https://www.gov.uk/government/publications/action-plan-for-animal-welfare/action-plan-for-animal-welfare.

[31] Rees P.A. (2005). Will the EC Zoos Directive increase the conservation value of zoo research?. Oryx.

[32] Mooney A., Conde D.A., Healy K., Buckley Y.M. (2020). A system wide approach to managing zoo collections for visitor attendance and in situ conservation. Nat. Commun..

[33] Marzella C. (2020). Safari Park Bosses Apologise After Monkey was Mauled to Death by Lion. Daily Record. https://www.dailyrecord.co.uk/news/local-news/safari-park-bosses-apologise-after-23184175.

[34] Mohamed E. (2021). Two Bears Shot at Whipsnade Zoo After Escaping from Enclosure. The Guardian. https://www.theguardian.com/world/2021/may/21/two-bears-shot-at-whipsnade-zoo-after-escaping-from-enclosure.

[35] Tozer J. (2021). Now Two Antelope are Shot After Fleeing Charity Linked to Carrie Johnson: Animals Were Gunned Down as They Escaped from Zoo Which Works with Foundation that Gave Job to Prime Minister’s Wife. Daily Mail. https://www.dailymail.co.uk/news/article-9690637/Now-two-antelope-shot-fleeing-zoo-run-charity-PMs-wife-Carrie-Johnson-works.html.

[36] BIAZA FAQs—How Does Zoo Licensing Work? 2026. https://biaza.org.uk/faqs.

[37] DEFRA (2019). Notice to Zoo Inspectors, Zoo Operators and Local Authoritie.

[38] Clegg I.L.K. (2018). Cognitive Bias in Zoo Animals: An Optimistic Outlook for Welfare Assessment. Animals.

[39] Sherwen S.L., Hemsworth L.M., Beausoleil N.J., Embury A., Mellor D.J. (2018). An Animal Welfare Risk Assessment Process for Zoos. Animals.

[40] von Fersen L., Encke D., Hütner T., Baumgartner K. (2018). Establishment and Implementation of an Animal Welfare Decision Tree to Evaluate the Welfare of Zoo Animals. Aquat. Mamm..

[41] Greenwood A., Cusdin P., Hicks S. (2003). Secretary of State’s Zoo Inspectors’ Performance.

[42] Davey G. (2007). Visitors’ Effects on the Welfare of Animals in the Zoo: A Review. J. Appl. Anim. Welf. Sci..

[43] Sherwen S.L., Hemsworth P.H. (2019). The Visitor Effect on Zoo Animals: Implications and Opportunities for Zoo Animal Welfare. Animals.

[44] Draper C., Browne W., Harris S. (2013). Do Formal Inspections Ensure that British Zoos Meet and Improve on Minimum Animal Welfare Standards?. Animals.

[45] Gardocka T. (2014). The Welfare of Animals in Zoos and EU Legal Standards.

[46] Scott P.W. (2017). Can zoo licensing be improved?. Vet. Rec..

[47] UK Government (2020). Sentencing Act. https://www.legislation.gov.uk/ukpga/2020/17/section/122.

[48] Brichieri-Colombi T.A., Lloyd N.A., McPherson J.M., Moehrenschlager A. (2019). Limited contributions of released animals from zoos to North American conservation translocations. Conserv. Biol..

[49] Gilbert T., Gardner R., Kraaijeveld A.R., Riordan P. (2017). Contributions of zoos and aquariums to reintroductions: Historical reintroduction efforts in the context of changing conservation perspectives. Int. Zoo Yearb..

[50] Drees K.V. (2003). The Evolving Mission of Modern Zoos and Aquariums: An Internal Appraisal.

[51] Zimmermann A., Kleiman D., Thompson K., Baer C. (2010). The Role of Zoos in Contributing to In Situ Conservation. Wild Mammals in Captivity: Principles and Techniques for Zoo Management.

[52] DEFRA Standards of Modern Zoo Practice for Great Britain: For Consultation. London, 2021. https://consult.defra.gov.uk/animal-health-and-welfare/1d5d9f40/supporting_documents/Standards%20of%20Modern%20Zoo%20Practice%20for%20Great%20Britain.pdf.

[53] ADAS (2011). Review of Local Authorities’ Implementation of the Zoo Licensing Act 1981 in England and Wales.

[54] Born Free Foundation (2026). Zoo Incidents Database.

[55] APGAW (2025). The Four Stages to Better Enforcement: Part 2 of Improving the Effectiveness of Animal Welfare Enforcement. London. https://apgaw.org/wp-content/uploads/2025/12/APGAW-The-Four-Stages-to-Better-Animal-Welfare-Report-2025.pdf.

