# Supplementary material for: Up to Standard? A Longitudinal Analysis of Regulatory Compliance in British Zoos
Source: Animals (Basel). 2026 Mar 28;16(7):1038. doi: 10.3390/ani16071038 (PMC13071974; doi:10.3390/ani16071038)
Supplement: Supplementary file 1 [file animals-16-01038-s001.zip › animals-4181999-supplementary.pdf]

**Supplementary Material**

Table S1. Zoo dispensation and exemption criteria.

Zoos which are either "traditional deer parks" or those which keep less than 120 specimens of "small, non-hazardous and non-conservation sensitive wild species" which do not consist of wild, mammalian species may be considered for an exemption under s.14(1)(a). Zoos keeping "non-hazardous and non-conservation-sensitive wild species, excluding wild mammals, not normally exceeding 200 specimens" may meet the requirements of a dispensation under s.14(1)(b). Whilst zoos which do not meet either of these requirements, but "the hazardous and/or conservation-sensitive species component of the collection not normally exceeds 50 specimens" may be able to receive a licensing dispensation under s.14(2).[3]

Table S2. Outcomes of sensitivity analysis.

| **Sensitivity Analysis (Reason)** | **Test Statistic** | **Change to Outcome** |
|---|---|---|
| Significant difference in the number of welfare criteria scored as substandard between first, second and third inspections (nine inspection forms missing Q.2.3 and Q.4.2 – recorded as a pass in original analysis) | (Friedman rank sum test, $\chi$ (2) = 21.14, $p < 0.01$) | No change |
| Significant difference in the number of welfare standards not met between second and third inspection (nine inspection forms missing Q.2.3 and Q.4.2 – recorded as a pass in original analysis) | (Wilcoxon signed ranks test, V = 1203, $p < 0.01$) | No change |
| Significant difference in the number of welfare standards not met between first and third inspection (nine inspection forms missing Q.2.3 and Q.4.2 – recorded as a pass in original analysis) | (Wilcoxon signed ranks test, V = 1333.5, $p < 0.01$) | No change |
| Significant difference in the number of welfare criteria scored as substandard between first, second and third inspections – BIAZA members (nine BIAZA zoos, not members at first inspection – recorded as BIAZA members in original analysis) | (Friedman rank sum test, $\chi$ (2) = 12.629, $p < 0.01$) | No change |
| Significant difference in the number of welfare criteria scored as substandard between first, second and third inspections – non-BIAZA members | (Friedman rank sum test, $\chi$ (2) = 10.231, $p < 0.01$) | No change |

| | | |
|---|---|---|
| (nine BIAZA zoos, not members at first inspection – recorded as BIAZA members in original analysis) | | |
| Significant difference in the number of welfare criteria scored as substandard between first, second and third inspections – EAZA members (two EAZA zoos, not members at first inspection – recorded as EAZA members in original analysis) | (Friedman rank sum test, $\chi$ (2) = 12.870, $p < 0.01$) | No change |
| Significant difference in the number of welfare criteria scored as substandard between first, second and third inspections – non-EAZA members (two EAZA zoos, not members at first inspection – recorded as EAZA members in original analysis) | (Friedman rank sum test, $\chi$ (2) = 12.156, $p < 0.01$) | No change |
| BIAZA zoos were significantly less likely to remain compliant with an individual welfare criterion than non-BIAZA zoos (nine BIAZA zoos, not members at first inspection – recorded as BIAZA members in original analysis) | (Fisher's exact test, OR = 0.636, 95% CI 0.532-0.760, $p < 0.01$) | No change |
| BIAZA member zoos scored as meeting significantly more of the conservation measures than non-BIAZA members (nine BIAZA zoos, not members at first inspection – recorded as BIAZA members in original analysis) | (Wilcoxon rank sum test, W = 7823, $p < 0.01$) | No change |
| EAZA member zoos scored as meeting significantly more of the conservation measures than non-EAZA members (two EAZA zoos, not members at first inspection – recorded as EAZA members in original analysis) | (Wilcoxon rank sum test, W = 3214.5, $p < 0.01$) | No change |

Table S3. Effect of collection type on the number of animal welfare criteria reported to be non-compliant by inspectors using a negative binomial Generalised Linear Mixed Model (GLMM).

| term | Rate_Ratio | CI_lower | CI_upper | P_value |
|---|---|---|---|---|
| (Intercept) | 1.605 | 0.644 | 3.999 | 0.310 |
| collection_typebird_of_prey | 1.053 | 0.302 | 3.669 | 0.936 |
| collection_typefarm_park | 3.618 | 0.905 | 14.470 | 0.069 |
| collection_typegeneral | 1.306 | 0.462 | 3.692 | 0.614 |
| collection_typeother | 0.912 | 0.176 | 4.710 | 0.912 |
| collection_typeother_bird | 0.483 | 0.085 | 2.729 | 0.410 |
| inspection | 1.159 | 0.788 | 1.702 | 0.454 |
| collection_typebird_of_prey:inspection | 1.280 | 0.752 | 2.180 | 0.363 |
| collection_typefarm_park:inspection | 0.880 | 0.488 | 1.587 | 0.671 |
| collection_typegeneral:inspection | 1.382 | 0.888 | 2.149 | 0.152 |
| collection_typeother:inspection | 1.193 | 0.600 | 2.372 | 0.615 |
| collection_typeother_bird:inspection | 1.429 | 0.688 | 2.966 | 0.338 |

Table S4. Difference in the number of zoos meeting conservation measures under s.1A of the ZLA across inspections.

| Conservation Measure | Cochran's Q Statistic (df) | p.value |
|---|---|---|
| (i) research from which conservation benefits accrue to species of wild animals | 7.600 (2) | 0.0224 |
| (ii) training in relevant conservation skills | 0.558 (2) | 0.756 |
| (iii) the exchange of information relating to the conservation of species of wild animals | 2.240 (2) | 0.326 |
| (iv) where appropriate, breeding of wild animals in captivity | 1.024 (2) | 0.599 |
| (v) where appropriate, the repopulation of an area with, or the reintroduction into the wild of, wild animals | 4.77 (2) | 0.0923 |

Table S5. Difference in the number of zoos meeting each conservation measure under s.1A of the ZLA across different paired inspections.

| Conservation Measure | Inspection Comparison | *p*.value | *p*.adj |
|---|---|---|---|
| 7.1 (i) | 1 vs 2 | 0.014 | 0.209 |
| 7.1 (i) | 2 vs 3 | 1.000 | 1.000 |
| 7.1 (i) | 1 vs 3 | 0.063 | 0.950 |
| 7.1 (ii) | 1 vs 2 | 0.850 | 1.000 |
| 7.1 (ii) | 2 vs 3 | 0.556 | 1.000 |
| 7.1 (ii) | 1 vs 3 | 0.860 | 1.000 |
| 7.1 (iii) | 1 vs 2 | 0.423 | 1.000 |
| 7.1 (iii) | 2 vs 3 | 0.239 | 1.000 |
| 7.1 (iii) | 1 vs 3 | 0.814 | 1.000 |
| 7.1 (iv) | 1 vs 2 | 0.458 | 1.000 |
| 7.1 (iv) | 2 vs 3 | 0.540 | 1.000 |
| 7.1 (iv) | 1 vs 3 | 1.000 | 1.000 |
| 7.1 (v) | 1 vs 2 | 0.584 | 1.000 |
| 7.1 (v) | 2 vs 3 | 0.170 | 1.000 |
| 7.1 (v) | 1 vs 3 | 0.074 | 1.000 |

Table S6. Model fit comparisons for Latent Class Analysis

| Class model | AIC | BIC | Log-Likelihood |
|---|---|---|---|
| 2 | 1680.134 | 1744.406 | -823.067 |
| 3 | 1671.040 | 1780.681 | -806.520 |
| 4 | 1749.784 | 1904.794 | -833.892 |

Table S7. Latent Class Analysis to estimate likelihood of participation in different conservation measures by zoo collection type.

| Collection Type | Class 1 | Class 2 | OR | 95% CI | Class 1 vs 2 *p*.value | Class 3 | OR | 95% CI | Class 1 vs 3 *p*.value |
|---|---|---|---|---|---|---|---|---|---|
| General Mixed (Baseline) | 93 | 45 | | | - | 0 | | | - |
| Aquarium | 30 | 20 | 0 | $(0, 3.7 \times 10^{36})$ | < 0.01 | 1 | $3.5 \times 10^{11}$ | $(3.5 \times 10^{11}, 3.5 \times 10^{11})$ | < 0.01 |
| Bird of Prey | 19 | 30 | $1.4 \times 10^{0}$ | $(7.1 \times 10^{-1}, 2.7 \times 10^{0})$ | 0.04 | 5 | $5.5 \times 10^{2}$ | $(5.5 \times 10^{2}, 5.5 \times 10^{2})$ | < 0.01 |
| Farm Park | 0 | 0 | N/A | N/A | N/A | 30 | N/A | N/A | < 0.01 |

| | | | | | | | | | |
|---|---|---|---|---|---|---|---|---|---|
| Invertebrate | 0 | 9 | N/A | N/A | < 0.01 | 0 | N/A | N/A | N/A |
| Other | 15 | 0 | N/A | N/A | N/A | 6 | $6.5 \times 10^{-1}$ | $(2.2 \times 10^{-1}, 1.9 \times 10^{0})$ | 0.2 |
| Other bird | 16 | 5 | $1.0 \times 10^{-2}$ | $(0, 1.0 \times 10^{-2})$ | 0.35 | 0 | N/A | N/A | N/A |
